# Costs and consequences of using average demand to plan baseline nurse staffing levels: a computer simulation study

**DOI:** 10.1136/bmjqs-2019-010569

**Published:** 2020-03-26

**Authors:** Christina Saville, Thomas Monks, Peter Griffiths, Jane Elisabeth Ball

**Affiliations:** 1 School of Health Sciences, University of Southampton, Southampton, Hampshire, UK; 2 University of Exeter Medical School, University of Exeter, Exeter, Devon, UK; 3 NIHR CLAHRC Wessex, University of Southampton, Southampton, Hampshire, UK; 4 Karolinska Institutet, Stockholm, Sweden

**Keywords:** simulation, nurses, health policy, health services research, decision analysis

## Abstract

**Background:**

Planning numbers of nursing staff allocated to each hospital ward (the ‘staffing establishment’) is challenging because both demand for and supply of staff vary. Having low numbers of registered nurses working on a shift is associated with worse quality of care and adverse patient outcomes, including higher risk of patient safety incidents. Most nurse staffing tools recommend setting staffing levels at the average needed but modelling studies suggest that this may not lead to optimal levels.

**Objective:**

Using computer simulation to estimate the costs and understaffing/overstaffing rates delivered/caused by different approaches to setting staffing establishments.

**Methods:**

We used patient and roster data from 81 inpatient wards in four English hospital Trusts to develop a simulation of nurse staffing. Outcome measures were understaffed/overstaffed patient shifts and the cost per patient-day. We compared staffing establishments based on average demand with higher and lower baseline levels, using an evidence-based tool to assess daily demand and to guide flexible staff redeployments and temporary staffing hires to make up any shortfalls.

**Results:**

When baseline staffing was set to meet the average demand, 32% of patient shifts were understaffed by more than 15% after redeployment and hiring from a limited pool of temporary staff. Higher baseline staffing reduced understaffing rates to 21% of patient shifts. Flexible staffing reduced both overstaffing and understaffing but when used with low staffing establishments, the risk of critical understaffing was high, unless temporary staff were unlimited, which was associated with high costs.

**Conclusion:**

While it is common practice to base staffing establishments on average demand, our results suggest that this may lead to more understaffing than setting establishments at higher levels. Flexible staffing, while an important adjunct to the baseline staffing, was most effective at avoiding understaffing when high numbers of permanent staff were employed. Low staffing establishments with flexible staffing saved money because shifts were unfilled rather than due to efficiencies. Thus, employing low numbers of permanent staff (and relying on temporary staff and redeployments) risks quality of care and patient safety.

## Introduction

Planning numbers of nursing staff on hospital inpatient wards is challenging because the demand for nursing staff is neither constant nor foreseeable, and to a lesser extent, neither is the supply. Demand on a single ward varies over time,^1^ for example, due to changing patient numbers, changing needs of those patients and changes in admission/discharge activity. Predicting demand before a shift is difficult, particularly on wards mainly dealing with unplanned admissions,^2 3^ although even planned admission lengths of stay and needs may vary due to variation in recovery times and surgical complications. Furthermore, nurses scheduled to work may be absent at short notice and opportunities for internal redeployment, overtime or hiring temporary staff to cover resulting shortfalls may be limited.

The method for determining the number of nursing staff allocated to a ward (the ‘staffing establishment’) is critical because it affects the number of nurses working each shift. Important elements of patient care are more likely to be omitted or delayed when nurse staffing is inadequate.^4^ Omissions range from vital patient surveillance and delayed response to deterioration^5^ to interpersonal care^6^ and there is growing evidence that these omissions adversely affect both patient safety and experience.^7–10^ For example, according to recent studies in England and the USA, a patient’s hazard of death in hospital increased by 2%–3% for each day of low registered nurse staffing.^11–13^ Furthermore, having too few nurses working on a shift is also associated with worse staff outcomes.^14^ On the other hand, having unnecessarily high numbers of nurses working on a shift could be a waste of a scarce resource; worldwide, nursing vacancy rates are high and registered nurses are in short supply.^15 16^

Nurse staffing tools and guidelines often recommend estimating the number of nurses needed each shift/day over a time frame, then taking the average to give a baseline staffing level for the ward.^17–20^ However, whether this will lead to having enough staff on the day is likely to be affected by how much demand varies from day to day and the availability of temporary staff to cover any shortfalls during peak periods.

In a simulation study involving stochastic optimisation, Harper *et al* 1 found that when more permanent nursing staff than the average needed were employed, the overall nurse staffing costs were lower. In a different context, Monks *et al* 21 highlighted the fallacy of supposing that planning stroke service capacity based on average occupancy is optimal. Instead, they recommended basing decisions on the trade-off between the simulated probability of delay in admission and the number of beds.

The impact of setting staffing levels at the average needed remains unclear. Existing modelling studies assessing nurse staffing decisions are limited by their use of small data sets (or assumptions) on how demand varies on wards. The question of whether these findings generalised is unanswered. Furthermore, existing studies neglect four practical issues that could affect staffing levels and costs.^1 17^ The first issue is the need for models to round to whole people/shifts (or realistic fractions) to reflect the reality of deploying staff, since staff time cannot be treated as continuously variable or divisible, allowing very small units of time to be allocated. Rather staff must be employed, deployed or moved for significant blocks of time (eg, minimum of 3 hours). The second shortcoming is the models neglect to include additional requirements for one-to-one care (known as enhanced care or specialing), which is increasingly important since some patient groups requiring specialing, such as those with severe mental health difficulties or at risk of falls, are growing.^22^ Third, existing studies assume that the relative productivity of staff is taken into account when requesting additional staff, when in reality it is likely that one nurse will be requested to cover for one absent nurse even if they are likely to be less familiar with the ward and therefore less productive. Finally, studies tend to assume that the estimate of demand made in advance is accurate while in fact patient needs are likely to vary from the plan.

In order to address this research gap, we use computer simulation to estimate the impact of different baseline staffing levels on understaffing and overstaffing rates, and on staffing costs in general wards of acute hospitals. This study aims to help policymakers and managers planning staff on wards, by exploring different strategies for setting staffing establishments in a safe, virtual environment, rather than testing them out on real wards with real patients. Unlike previous simulation studies of nurse staffing decisions,^1 23–27^ we incorporate a range of practical issues and make use of large data sets, in particular for the variation in need on each ward. Our objectives are, first, to compare simulation results (understaffing rates, overstaffing rates and costs) when baseline staffing levels are set at the average needed, versus lower and higher levels, and second, to investigate the effects of flexible staffing to respond to variation in demand, assuming different degrees of temporary staff availability.

## Methods

Using workforce and patient data, we developed a Monte Carlo simulation of nurse staffing on general hospital wards. We simulated the varying demand for nursing care (measured by the ‘Safer Nursing Care Tool’ (SNCT))^18^ and varying available staffing levels. We tested different strategies to meet the demand and compared their staffing costs and understaffing/overstaffing rates.

### Study setting

To provide parameters for our models, we collected data in the adult medical/surgical inpatient wards of four acute hospital Trusts (hereafter referred to as hospitals), consisting of one university hospital, two district general hospitals and one specialist cancer hospital based in London, South East and South West England. We excluded highly specialised services and day case units. Our data set included 81 wards with 2178 beds (74% of all beds in the study hospitals). Wards in these hospitals used a variety of shift patterns with variation between and within wards in the mixture of long (12+ hours) and shorter shifts. Data were gathered over the course of a year (2017) in order to provide robust estimates of all model parameters, and sufficient observations for each ward from which to sample the numbers of patients and their acuity/dependency levels.

### Outcome measures

We calculated the number of ‘patient shifts’ (the sum of patients occupying beds on wards at the start of each shift) that were understaffed/overstaffed by more than 15%, relative to the actual staffing requirement for each day. The tolerance of 15% corresponds to that used in the RAFAELA staffing tool.^28^ The achieved staffing takes account of reduced productivity of redeployed and temporary staff and the actual staffing requirement accounts for patient needs varying from the typical need for their acuity/dependency category.

We also calculated the cost per patient-day from the total annual staffing cost, including both permanent and temporary staff across all wards, as well as payments for working unsocial hours. This uses the band (grade) mixes of permanent staff as actually worked on each ward, and the band mixes of temporary staff as worked across each hospital.

### The simulation model

Our model simulated variation in the demand for nurses as measured by the SNCT for each shift on each ward on each day. We also simulated the staff deployed on the ward after unexpected absences, internal redeployment and deployment of temporary staff from the internal bank or external agencies. For each shift for each ward the model sought to fill any staffing shortfalls, first by redeploying staff from wards with an excess of staff (subject to constraints about whole people who must be redeployed for a half/whole shift). Where staff were not available from other wards, temporary staff were deployed, subject to constraints on the availability of temporary staff at short notice.

**Figure 1:**
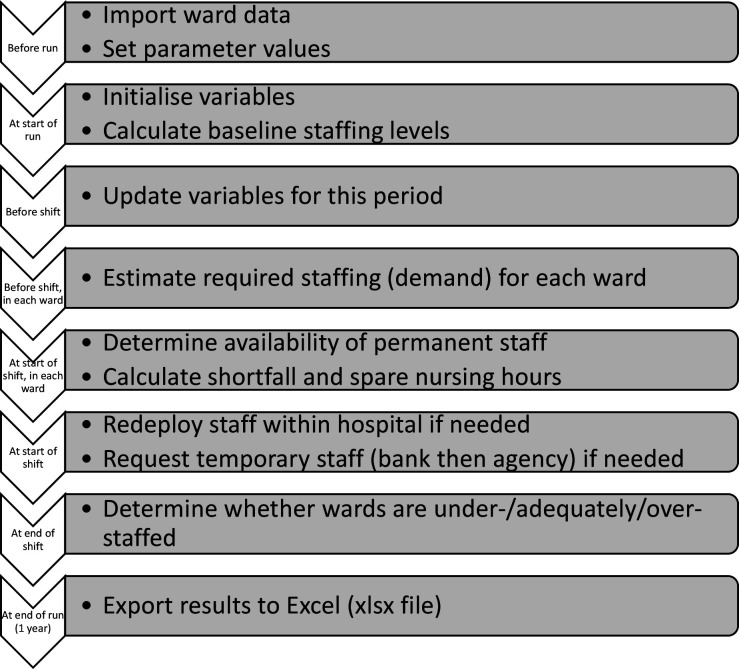
Flow chart of simulation steps.

The model is an agent-based simulation (ABS) of a hospital with a flexible number of wards, specified by the user. These wards are the ‘agents’, which move between being understaffed, adequately staffed or overstaffed (their ‘states’) each shift. We developed the simulation in AnyLogic software. It is an example of a Monte Carlo simulation since many input parameters (such as absence rates and demand for nurses) are stochastic so are modelled as random variables following probability distributions. Figure 1 shows the main simulation steps, and both a video of the simulation in action^29^ and a detailed model description following the Strengthening The Reporting of Empirical Simulation Studies (STRESS) reporting guidelines for agent-based simulation (ABS)^30^ (see online supplementary material) are available.

10.1136/bmjqs-2019-010569.supp1 Supplementary data

### Staffing scenarios

In our primary experiments, we investigated the impact of different baseline staffing levels on understaffing/overstaffing and costs. To calculate these staffing levels, we used daily records of patient demand for nursing care, as assessed by the nurse in charge using the SNCT. This is a patient classification tool, which works by categorising patients according to their acuity and dependency on nursing care.^18^ The five categories (levels 0, 1a, 1b, 2 and 3) range from patients needing normal ward care to those needing advanced respiratory support/therapeutic support of multiple organs. According to a recent survey, this tool is used to guide staffing decisions in most acute National Health Service (NHS) hospitals in England^31^ and is also used in Canada and New Zealand.

For the primary experiments, we combined the daily demand across an observation period in different ways to obtain the total number of nursing staff to employ on a ward (see table 1). The observation period to determine baseline staffing establishments recommended in the current version of the SNCT guidelines is 20 days twice a year in January and in June,^18^ and we used June to avoid any peculiarities relating to new year. We converted the number of staff to employ into a daily measure (nursing hours per day). We accounted for skill mix by splitting staffing levels between registered nurses and assistants according to the observed skill mix on each ward (the SNCT advises using professional judgement for the skill mix so this is a proxy). To account for variation between shifts, we calculated the planned morning/afternoon/evening and night staffing by distributing staff over four 6-hour periods, corresponding to the mixed pattern of 12 hours and short shift systems used and according to observed patterns in staffing levels over 24-hour periods. We rounded requirements to the nearest multiple of 4/6 hours to recognise that staff can only be requested for fixed time blocks. We based the availability of temporary staff on empirical data provided by one of the participating hospitals. Here, the chance of bank/agency staff being available to cover at short notice was between 5% and 45% depending on the time of day and staff type.

**Table 1:** Staffing scenarios

Primary experiments—baseline staffing levels	
Standard (core assumption)	Average demand measured by the Safer Nursing Care Tool (SNCT) across 20 days in June* as recommended by the tool guidelines.
Low	80% of average demand measured by the SNCT across 20 days in June*. Lower than the standard either due to vacancies or set to provide bare minimum coverage with high use of flexible staffing.
High	90th percentile of demand measured by the SNCT across 20 days in June*. Designed to meet demand on most days—it is theoretically enough to meet demand through permanent staff on 90% of days.

Secondary experiments—flexible staffing options	
Empirical availability (core assumption)	Redeployments and empirical availability of temporary staff (<50% chance of fulfilled requests for all staff types and times of day). Based on data for temporary staffing request fulfilled for one of the participating hospitals.
No temporary staff or redeployments	No redeployments and no temporary staff.
Higher availability	Redeployments and bank/agency staff requests each have 50% chance of being fulfilled. Thus, this assumes higher availability of temporary staff than the core assumption.
Unlimited availability	Redeployments and bank staff requests each have 50% chance of being fulfilled and agency staff requests have 100% chance of being fulfilled.

*Or next month if unavailable. Where needed for wards that have no data, for example, in the second half of the year, we applied the average percentage increase/decrease between January and June for other wards at that hospital.

In our secondary experiments, we investigated the impact of different degrees of flexible staffing (see table 1). We considered the following types of flexible staffing. As a first response to a shortage (assessed relative to the SNCT estimate for the patients present on that shift), attempts are made to redeploy staff between wards in the same speciality-specific division. If shortages persist, internal bank and then external agency staff are requested. In all cases, registered nurses are substituted for registered nurses, and assistants are substituted for assistants. Evidence suggests that temporary and redeployed staff may be associated with worse care quality and outcomes,^32^ so we assumed they were less productive than permanent staff, with redeployed and bank staff being 90% as efficient, and agency staff being 75% as efficient.

### Data sources

The data sources consisted of actual worked rosters (for skill mixes and the distribution of staff over the day), patient admissions/transfers/discharges (for numbers of patients), SNCT ratings (for proportions of patients in each acuity/dependency level) and specialed patients’ reports (for numbers of patients requiring one-to-one or cohort specialing). Administrative patient data on ward admissions and discharges are updated by ward staff and bed managers. SNCT ratings are undertaken by nurse shift leaders and recorded in near real time on a dedicated system, which is used to monitor acuity across the hospitals. Roster data are input in advance by ward managers and verified/updated to reflect changes on the day, including recording staff absences and temporary staff hires.

We used national reference costs^33^ for the unit costs of staff in each pay band. We assumed that employers pay superannuation payments for 50% of bank staff (senior nurses agreed this was a reasonable assumption), making their hourly rate slightly cheaper than for permanent staff in the same pay band. Agency costs were based on agency caps^34^ and unsocial hour rates were those in use at the time of the study.^35^

### Model parameters, run length and replications

In the simulation, we modelled the variability inherent in various aspects of the nurse staffing process by sampling from empirical probability distributions. Proportions of patients in each acuity/dependency level were sampled once per 6-hour shift from empirical data for each ward. For the numbers of patients, we did the same but with different distributions for each day of the week. Current national sickness rates for nursing staff are 4.5%,^36^ so we assumed rates of unanticipated absence through short notice sickness of 4% for assistants and 3% for registered nurses, approximating known differences in sickness absence between these groups.^37^ The probability of bank and agency staff requests being fulfilled was based on different assumptions regarding the availability of these staff (see table 1).

The model was run to simulate data for a period of 1 year because hospital finances are typically planned over a year. We ran the model 10 times (ie, for 10 independent years) for each hospital and for each staffing scenario, and calculated 95% CIs around the means to assess the errors around the estimates. We chose this number of replications because it gave 95% CIs that were narrow (for the standard staffing scenario, widths were <£0.25 cost per patient-day and <0.7% understaffed patient shifts at all hospitals) with manageable computation times (<1 hour per model). Given the narrow CIs, we report means only.

### Model verification and validation

Throughout model development, we performed verification (checking correct implementation of the model in simulation software) and validation (checking that we built an appropriate and accurate enough model). As recommended by Sargent,^38^ we documented these checks (see online supplementary material). In particular, we worked closely with nurses with responsibilities for workforce at the four hospitals, who agreed assumptions and sense-checked results. We also presented and discussed early versions of the simulation model and results with the project steering group, which included nursing research, mathematical modelling and nursing workforce experts.

The results of the models are not directly comparable with the experiences of the hospitals that we derived the data from because they may not always use the SNCT ratings to guide their decisions of baseline staffing, which may be constrained and altered by staff availability, whereas our models were mostly based on the assumption that hospitals could employ the establishments that they required, only using temporary staff to make up shortfalls. The level of temporary staff actually used in these hospitals was consequently generally higher than in our models (table 9 in our report^39^) as were the staff costs. However, estimated daily staff costs were broadly similar (£140–£150 per patient-day (data in table 15^39^)) except in the specialist hospital whose actual costs were much higher than we estimated. In this hospital it is recognised that the establishments deployed are very high relative to those calculated by the SNCT.

## Results

### Sample sizes and planned baseline staffing levels

‘Standard’ baseline staffing levels were on average 155, 207, 78 and 171 total (registered nurse plus assistant) hours per day across wards at hospitals A, B, C and D, respectively, varying between wards from 42 to 576 hours per day. Patient numbers varied, but on average across all wards, standard baseline staffing levels corresponded to 6.7 hours per patient-day (see table 2). The skill mixes (percentage of daily hours provided by registered nurses) were 51%, 56%, 75% and 49% on average across wards at hospitals A–D.

**Table 2:** Characteristics of the study hospitals

Hospital	Wards	Ward shifts	Patient shifts	Ward beds			Baseline staffing (hours per patient-day)—mean per ward		
	n	n	n*	Mean	Min	Max	Low	Standard	High
A	19	27 759	632 060	23	8	42	5.5	6.8	7.5
B	31	45 291	1 361 430	29	10	63	5.5	6.9	7.3
C	12	17 532	210 492	14	8	21	5.5	6.5	7.7
D	19	27 759	719 632	27	18	36	5.2	6.6	7.1
All	81	118 341	2 923 614	25	8	63	5.4	6.7	7.3

*Varies between runs since the number of patients is random sample of what is expected in a typical year. This is the average across 10 runs for the core scenario (empirical availability and standard staffing).

By construction, ‘low’ baseline staffing levels were approximately 80% of standard levels, with slight differences due to rounding whole bodies/shifts. The low baseline staffing levels corresponded to 5.4 nursing hours per patient-day on average across all wards. ‘High’ baseline staffing levels were on average 10% higher than standard levels. These high baseline staffing levels corresponded to 7.3 nursing hours per patient-day on average across all wards.

### Primary experiments: alternative baseline staffing levels

We start by reporting simulation results for the whole sample, assuming the empirical availability of temporary staff (<50%), and then explore hospital-level and ward-level variations.

For all baseline staffing levels tested, understaffing occurred relatively often, despite using flexible staffing. When baseline staffing was set to meet the average needed (the standard approach), 32% of patient shifts were understaffed (more than 15% hours short). When baseline staffing levels were ‘low’ (10% lower than standard levels), the understaffing rate was substantially higher, at 65% of patient shifts. Understaffing rates were reduced to 21% of patient shifts for ‘high’ baseline staffing levels (theoretically enough to meet observed demand on 9 out of 10 days).

Overstaffing (more than 15% surplus hours) occurred relatively rarely for all baseline staffing levels tested. With standard staffing levels, 4% of patient shifts were overstaffed, while with low staffing levels, less than 1% of patient shifts were overstaffed. With high staffing levels, just under 10% of patient shifts were overstaffed. For all the baseline staffing levels, staff were rarely moved between wards (see online supplementary appendix table), because overstaffing is relatively rare and redeployment can only occur when understaffing is matched with overstaffing within the redeployment pool. Of the total hours worked, 0.2%, 0.6% and 0.9% were worked by redeployed staff under low, standard and high baseline staffing levels, respectively.

10.1136/bmjqs-2019-010569.supp2 Supplementary data

The higher the baseline staffing level, the fewer hours were worked by temporary staff. Of the total hours worked, 16%, 7% and 5% were worked by temporary staff under low, standard and high baseline staffing levels, respectively. Total staffing costs were higher for higher baseline staffing. The staffing costs were on average £120, £135 and £142 per patient-day for low, standard and high staffing levels, respectively.

The pattern of results was the same at all hospitals; the higher the baseline staffing level, the fewer patient shifts were understaffed and the more patient shifts were overstaffed (see table 3). However, the rates of understaffing and overstaffing differed between hospitals. At all hospitals, under low baseline staffing, more than 55% of patient shifts were understaffed, while under high baseline staffing, fewer than 30% of patient shifts were understaffed. Differences between hospitals reflect ward-level differences in the impact of different baseline staffing levels. The costs also differed between hospitals, due to the different skill and band mixes in their wards, as well as different degrees of temporary staff use. Hospital A had the lowest costs in all scenarios while hospital C had the highest.

**Table 3:** Variation in understaffed/overstaffed patient shifts and costs between hospitals, for different baseline staffing scenarios, assuming empirical availability of temporary staff

Baseline staffing	Low	Standard	High	Low	Standard	High	Low	Standard	High	Low	Standard	High
Hospital	Patient shifts			Understaffed			Overstaffed			Cost per patient-day		
	n	n	n	%	%	%	%	%	%	£	£	£
A	632 316	632 060	632 256	55.7	17.8	7.5	1.0	8.8	18.6	110	125	136
B	1 361 556	1 361 430	1 361 442	69.9	36.1	26.4	0.3	2.2	5.3	125	138	144
C	210 644	210 492	210 648	60.4	37.4	16.2	4.6	13.0	30.7	130	148	169
D	719 490	719 632	719 741	66.8	34.4	25.0	0.2	2.3	4.7	119	132	137
Whole sample	2 924 006	2 923 614	2 924 087	65.4	31.8	21.2	0.7	4.4	9.9	120	135	142

Average results across 10 runs of the simulation model.

For all hospitals, the 95% CIs were less than 1%/£0.50, so not reported.

Understaffing varied considerably at a ward level (understaffed patient shifts ranged from 7% to 80% under the ‘standard’ staffing scenario) but less variation in overstaffing (corresponding range 0%–35%). The percentage of shifts that were overstaffed and the percentage of shifts that were understaffed were moderately negatively correlated (r=−0.460; p<0.01).

### Secondary experiments: use of flexible staff

The assumed availability and use of flexible staff to meet demand affected the understaffing rate and costs substantially. For all baseline staffing levels, as more temporary staff were available to cover at short notice, costs increased while the rate of understaffing decreased (see table 4). The difference between baseline staffing scenarios reduced the more temporary staff were available, both in terms of costs and understaffing. Even with unlimited temporary staff, the low baseline staffing strategy still led to considerably more understaffed patient shifts (>17%) than higher baselines (8% or less), while cost differences between scenarios were substantially reduced, with the high staffing scenario costing £7 per patient-day more than low staffing. This pattern in understaffing is because more temporary staff worked when baseline staffing was low and temporary staff were assumed to be less productive. The cost per patient-day was lowest when flexible staffing was not used, but in this case staffing was poorly matched to demand; both understaffing and overstaffing were high. Overstaffing was highest when there was no flexible staffing; with flexible staffing, attempts were made to move surplus staff elsewhere and temporary staff were only requested to raise staffing levels to ‘adequate’ so did not contribute to overstaffing.

**Table 4:** Understaffed/overstaffed patient shifts and costs by temporary staff availability for different baseline staffing levels

Baseline staffing	Low	Standard	High	Low	Standard	High	Low	Standard	High	Low	Standard	High
Flexible staffing option	Patient shifts			Understaffed patient shifts			Overstaffed patient shifts			Cost per patient-day		
	n	n	n	%	%	%	%	%	%	£	£	£
Unlimited availability	2 924 099	2 923 561	2 923 896	17.1	8.0	4.4	0.7	4.5	9.9	145	147	152
Higher availability	2 923 631	2 924 193	2 923 489	41.1	18.2	11.5	0.7	4.4	9.9	135	142	148
Empirical availability	2 924 006	2 923 614	2 924 087	65.4	31.8	21.2	0.7	4.4	9.9	120	135	142
No temporary staff or redeployments	2 923 940	2 923 668	2 923 786	86.0	46.6	33.8	1.0	5.8	12.4	100	124	135

Calculated from average results across 10 runs of the simulation model for each hospital.

## Discussion

Our simulation model has demonstrated that setting baseline staffing to meet average demand is associated with high levels of understaffing even if unlimited use of flexible staffing to meet need is assumed. While a lower baseline staffing level is associated with lower costs, these lower costs arise almost entirely due to leaving shifts unfilled. If unlimited availability of temporary staff is assumed, the risk of understaffing is reduced but it remains relatively high, with cost savings substantially reduced. Higher baseline staffing, set to meet the demand observed on 90% of occasions, is associated with much lower risk of understaffing with modest increases in cost when extensive use is made of flexible staffing.

The fallacy of using averages for planning has been documented elsewhere,^21^ yet planning nurse staffing levels to meet average demand is still widespread in nursing tools and in national guidance for safe nurse staffing levels.^18 19^ In contrast, both the Royal College of Physicians’ guidance for medical staffing^40^ and the Royal College of Midwives’ guidance for midwifery staffing on maternity wards^41^ recommend that staffing should be set at 80% of maximum demand. The midwifery staffing guidance recognises that this could be tailored up or down, with higher figures requiring less use of flexible staff. Setting baseline staffing levels at the average observed demand means that even before accounting for issues such as rostering/rounding, unavailability of staff and additional one-to-one care demands, staffing will only be sufficient on days with close to average or lower than average demand. We observed that the staffing requirement on many wards (one out of three) had a left-skewed distribution; on these wards mean average staffing demand is likely to be exceeded more than 50% of the time. Our simulation results suggest higher than average staffing giving better fit to need in terms of fewer understaffed patient shifts. Overstaffing, while potentially costly, was relatively rare.

The availability of temporary staff who can work at short notice greatly affects the results, particularly at lower baseline staffing levels when they are requested more often. The greater the availability, the less difference between high, standard and low baseline staffing scenario results. In particular, with unlimited temporary staff (needed for the low baseline staffing level to be feasible in terms of staffing adequacy), the cost savings compared with higher staffing levels are smallest. Rather than employing more staff, an alternative approach could be to find ways to increase the chance of temporary staff being available at short notice. However, cost savings might be negated through additional payments, for example, higher rates for on-call/stand-by staff or unpopular shifts.

Under our core assumption for the availability of temporary staff, flexible staffing with low baseline staffing exposes patients to more than 50% understaffed patient shifts at all four included hospitals. The high rate of understaffing occurs despite redeploying staff between wards and requesting temporary staff to cover when needed. This is because under low baseline staffing levels, wards rarely have surplus staff to redeploy elsewhere. Furthermore, according to empirical data from hospital B, the chance of a request for bank/agency staff being fulfilled at short notice is less than 50%, with the chance being as low as 5%–10% for some staff types and time periods. Although cost savings are apparent when lowering baseline staffing levels, this is largely due to having too few staff working on the day, rather than only reducing cases of surplus staff.

In contrast, flexible staffing coupled with high baseline staffing levels is a more promising approach. Setting baseline staffing at levels high enough to meet measured demand on 90% of days means that flexible staff, who were assumed to be less productive, are only used to cover shifts with the highest demand. The simulation results highlight the importance of employing enough staff, as opposed to relying on flexible staff as a ‘magic bullet’ in times of workforce shortages. Under our core assumptions about flexible staffing, the additional cost of high baseline staffing over standard baseline staffing is £7 per patient-day, but these costs do not account for potential savings of reduced bed-days or improved patient outcomes associated with the substantially reduced risk that patients experience understaffing.^42 43^

For patients, there are multiple quality and safety consequences of being cared for on understaffed wards. Having enough staff is, in itself, a key measure of quality; the publication of ‘fill rates’ by the NHS in the past is an acknowledgement of this.^44^ A recent review found 14 studies linking low nurse staffing levels with omissions of nursing care provided to patients.^4^ A systematic review in 2007 showed the link between higher registered nurse staffing levels and lower risks of a range of adverse patient outcomes including pneumonia acquired in hospital, cardiac arrest, respiratory failure and hospital-related mortality.^9^

### Comparison with previous studies

Harper *et al* 1 found that total nurse staffing costs were lower when more permanent nursing staff than the average needed were employed. Their assumptions correspond most closely with our ‘unlimited temporary staff’ scenario but they assumed that all demands would be met, that is, more flexible staff would be deployed to do the job of one permanent member and the true demand was the same as what was planned for. Therefore, it was unnecessary to compare understaffing rates between scenarios.

In contrast, we found the opposite: setting baseline staffing at the average demand was cheaper than setting it at higher levels, with a further smaller cost saving achieved by setting levels lower. This shows the sensitivity of results to assumptions and unit costs. Like Harper *et al*,^1^ our results also lead us to recommend higher staffing levels but for reasons of reducing understaffing rather than direct cost savings, although as temporary staffing availability increases, our results converge.

### Strengths and limitations

This study helps bridge the gap between the existing, mainly theoretical, modelling studies^17^ and practical questions around nurse staffing, by incorporating real-world issues and using extensive empirical data. Collaborating with nurse staffing research specialists and nurse managers highlighted practical issues to include in the simulation model, so was essential for the validity and usability of the results. In particular, the model accounted for the unpredictability of the true staffing requirement each shift (patients’ needs can vary from the average for their acuity/dependency category), ability of staff to adapt to workload variation (buffer around staffing requirements each shift), limited availability of temporary staff, one-to-one and cohort care requirements, rostering limitations (staff are deployed for 4 or 6-hour time blocks) and the fact that at the time of requesting, temporary staff are assumed to be as efficient as permanent staff. As far as we can ascertain, other simulation studies have not addressed these factors.^1 23–27^ The focus of previous studies has tended to be on developing new models/methods and demonstrating their use on data sets for single wards/single hospitals. Although we also developed a new model, our focus was on generating findings relevant to more than one place, so we conducted a large study on linked data from 81 wards in four hospitals. By collecting data across multiple settings, we showed that patterns were consistent and not unique to one hospital.

Our modelling included a number of assumptions. We assumed there is no interaction between the demand for nursing in different wards and that time periods are independent of one another. Any such dependencies would likely exacerbate staff shortages, making redeployments less likely. Although the effect of this assumption remains untested, the contribution of internal redeployments to remedy staffing shortfalls was relatively small and so overall results are unlikely to change dramatically. We only modelled rostering processes indirectly, by rounding nursing hours. Our particular assumptions about the relative productivity of temporary staff were untested, but our sensitivity analysis suggested that this assumption made little difference to the overall estimate of understaffing,^39^ although recent studies highlight the potential that supervision of temporary staff may reduce the productivity of the team as a whole.^32^ We used data from four hospitals in England and saw consistent patterns, but the results may not transfer to other settings.

### Implications

Based on the results of this study we recommend that, as in other fields,^40 41^ planning to the average should cease to be the default position in nurse staffing tools. Our study demonstrates the importance of flexible staffing but the results challenge the assumption that a low baseline staffing establishment with heavy use of flexible staffing is either feasible, effective or efficient. Rather, the priority is to ensure high baseline staffing levels, for example, sufficient to meet ward needs on 90% of days. Flexible staffing can be used to make minor adjustments to staffing levels on the day.

In order to be more useful to nurse managers, we recommend that models looking at other nurse staffing decisions consider more practical issues and how model results differ between settings.

This study focused on hospital-level understaffing rates, but this hides differences between wards, which warrant further investigation.

## Conclusions

While it is common practice to base staffing establishments on average demand, our results suggest that this may lead to more understaffing than setting establishments at higher levels. Flexible staffing, while an important adjunct to the baseline staffing, was most effective at avoiding understaffing when high numbers of permanent staff were employed. Low staffing establishments with flexible staffing saved money because shifts were unfilled rather than due to efficiencies. Thus, employing low numbers of permanent staff (and relying on temporary staff and redeployments) risks quality of care and patient safety.
