# Supplementary material for: Costs and consequences of using average demand to plan baseline nurse staffing levels: a computer simulation study
Source: BMJ Qual Saf. 2020 Mar 26;30(1):7–16. doi: 10.1136/bmjqs-2019-010569 (PMC7788209; doi:10.1136/bmjqs-2019-010569)
Supplement: Supplementary data [file bmjqs-2019-010569supp001.pdf]

Supplementary Material

Reproduced from: Appendix 1 in Griffiths P, Saville C, Ball J E, Chable, R, Dimech A, Jones J, Jeffrey Y, Pattison N, Recio Saucedo A, Sinden N and Monks T. Crown copyright 2019. Identifying nurse-staffing requirements using the Safer Nursing Care Tool. Modelling the costs and consequences of real world application to address variation in patient need on hospital wards. Health Services and Delivery Research [In press]. Changes to original: abbreviations spelled out and cross-references amended.

Simulation model technical details

Implementation and experimentation

The simulation was developed on a Windows 10 Enterprise operating system version 1709, build number 16299.726 in Anylogic 8 researcher edition software version 8.3.2, build number 8.3.2.201807061745 x64. This used Java 2 Standard Edition 8.0 and Fluid library version 2.0.0.

We used the default random number generator in Anylogic: an instance of the Java class Random, which is a Linear Congruential Generator (LCG).

Time is modelled as fixed time steps. There are no concurrent events that are interdependent.

There is no warm-up period or model initialisation since each day/shift is independent of the next. The model is stochastic. The run length is one year, and the time units are six-hour shifts (the model also works for days). The number of replications is 10.

Running one experiment (1 year, 10 runs) takes between 10 and 20 minutes for the different hospital Trusts on a Dell Latitude laptop with Intel® Core™ i5-7300U processor with 2.60GHz CPU speed, 8 GB RAM and 32 GB ROM.

Computer Model Sharing Statement

For a video demonstration see https://eprints.soton.ac.uk/430632/. The simulation model is available from the corresponding author on reasonable request. Anylogic simulation software can be downloaded from https://www.anylogic.com/downloads/.

Simulation model logic

An overview of the simulation logic is shown in Figure 1. Each stage is described in more detail below. The simulation was designed to work for any number of wards. The wards are ‘agents’ which interact with each other (within directorates) by lending/borrowing staff in an attempt to cover shortfalls of staffing in one ward with surplus in another. Outputs are updated at the end of each time step (day/shift) and aggregated at the end of each year.

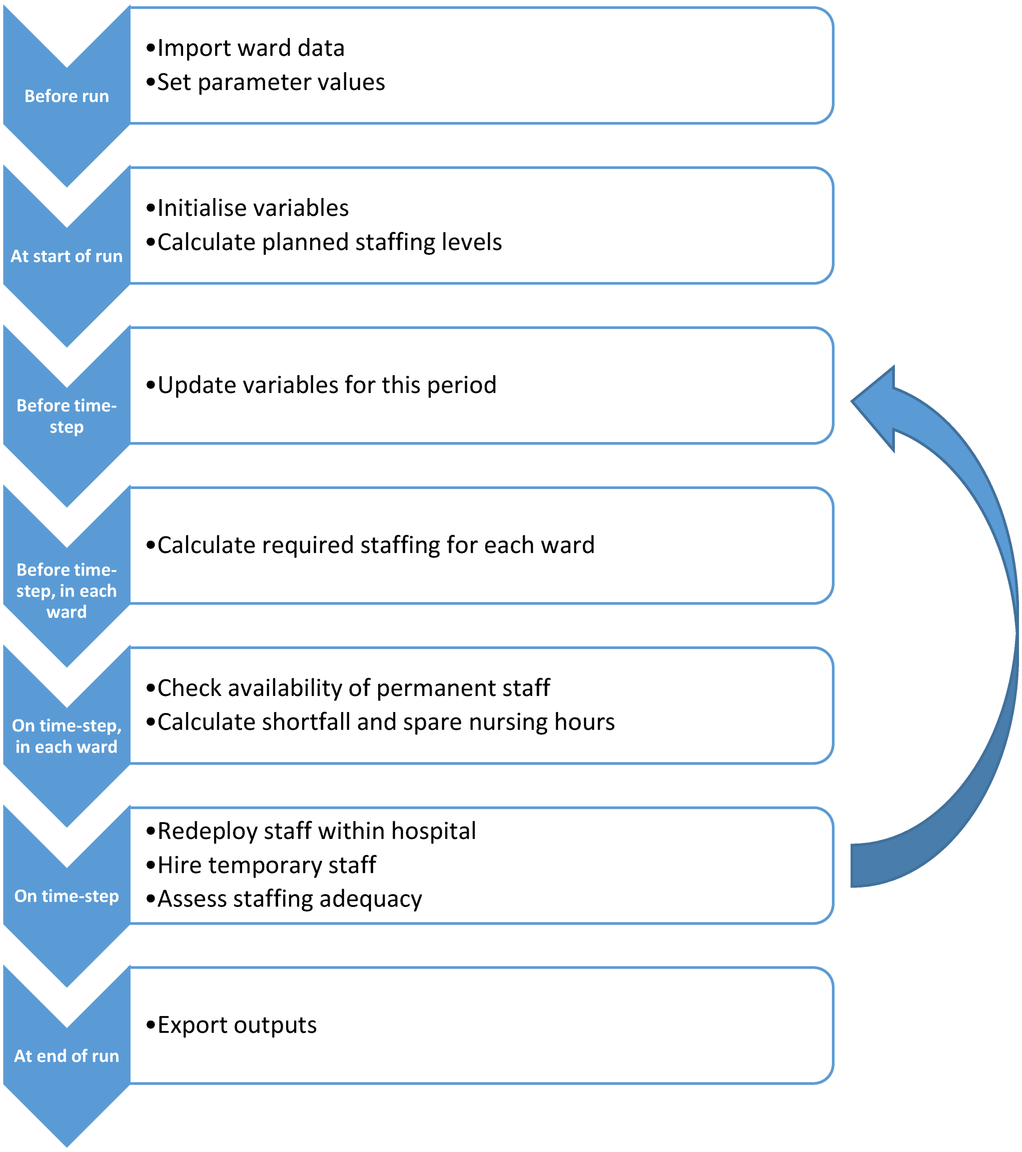

FIGURE 1 **Simulation model overview diagram**

Before running the simulation

Before running the simulation for a particular hospital Trust, information about its wards is imported from Excel files to fill database template tables within the simulation. The three files contain occupancy data, acuity/dependency data and other ward information. The occupancy distributions consist of the number of patients, labelled with the ward identification numbers (a contiguous number for internal purposes and the number used for external reporting), day of the week and 6-hour-shift. The acuity/dependency distributions consist of the proportions of patients in each SNCT acuity/dependency level and the proportion of patients that require specialing, labelled with the ward identification numbers and observation number (used when sampling a row). The other ward information consists of the baseline staffing levels for each scenario (in WTE), the skill mix (in the morning, afternoon, evening, night, and over a 24-hour period), the distribution of staff over 24

hours (proportion of staff deployed in the morning/afternoon/evening/night), the directorate number, whether the ward is an acute admissions unit and some purely descriptive information (directorate, medical or surgical).

The probabilities of requests for temporary staff being fulfilled is imported from an Excel file into another database template table. The template allows probabilities to differ between bank and agency, up to three staff types, weekend and weekday, and time period (morning, afternoon, evening, night, and over a 24-hour period).

Next, further parameter values that apply globally to the whole hospital Trust (the system) are set. These parameters relate to:

(1) nursing staff requirements - acuity/dependency multipliers, specials multiplier, standard deviation of multipliers, minimum number of registered nurses needed (constraint), demand for registered nurses with a particular skill

(2) permanent staff - the baseline staffing level, the data sample used to calculate the staffing level, the staff types, absence chance for each staff type, absence length, proportion of registered nurses with a particular skill

(3) redeployed staff - redeployment rules, priority sequences for providing and receiving redeployed staff, efficiency of redeployed staff, redeployed staff shift length

(4) temporary staff - rules for requesting temporary staff, efficiency of bank and agency staff, temporary staff shift length

(5) display settings - the understaffing criterion to plot in charts, e.g. 15% or more under requirement

(6) general settings - step length, round down bound used when converting required hours to requested hours.

At start of run

At the start of a run, variables tracking time (the step number and the shift number) are reset to zero. The wards, staff types and sharing groups are counted. Occupancy distributions are created from the occupancy data table. Data are placed in arrays, which are convenient structures for working with multi-dimensional data.

Then, the establishment (number of staff employed in WTE) is converted to the number of planned deployed hours per time step (6-hour-shift or day), including applying the skill mix, rounding to whole people and dealing with minimum constraints, as follows. Note that the establishment does not need to be a whole number since staff may work part-time, but the planned number of staff to deploy each six-hour shift should be a whole number. As in our other analyses we use equation 3 for converting the planned staffing in WTE to the planned total care hours per day (see Equation 3, Appendix 1, main report[1]).

The planned skill mix (proportion of staff that are registered nurses) and distribution of staff over the day in each ward is set as the average observed for that ward. There is a constraint that there must be at least one registered nurse present on each ward, so if the registered nurse hours is under 6, this is rounded up to 6. Otherwise, the registered nurse hours are rounded up or down to the nearest six hours. The remaining planned hours are assigned to nursing support workers, and again rounded up or down to the nearest 6 hours.

For example, suppose the planned nursing hours for a morning shift on a particular ward are 18, and the skill mix is 50%. This is equivalent to 9 hours of registered nurse time, which is rounded up to 12 hours. There are 6 hours left to cover which are assigned to nursing support workers.

The planned deployed hours per day (sum over the four shifts) are converted back into WTE to enable calculation of the cost of employing this number of permanent staff.

Before time-step

Before each time-step, i.e. before the simulation switches to the next period (six-hour shift or day), the variables for this period are updated. These variables are the time step, the shift (1 to 4), the day type (weekday, Saturday of Sunday/bank holiday) and the planned staffing level for this shift. The deployment array (numbers of staff from each source and of each staff type deployed on each ward in what capacity) is reset at zero ready to be filled in the next stages.

Before time-step, in each ward

Next, the required staffing for this period is calculated for each ward in turn. For this, firstly the number of patients on the ward is sampled from the occupancy distribution for that ward, day of week and shift (morning, afternoon, evening or night).

Secondly, the acuity/dependency profile is sampled from the acuity/dependency data. This is done by selecting a random observation for that ward (we assumed there were no day of week or time of day patterns). For each patient, the probability of being in each acuity/dependency category and the probability of requiring specialing are the corresponding observed proportions. The required staffing per patient (in WTE) is sampled based on the patient's acuity/dependency category and specialing requirements. This is converted into the required staffing level for this period using the skill mix, distribution of staff over the day and minimum constraints (as for the planned staffing levels), but is not rounded.

On time-step, in each ward

On the time-step, i.e. immediately when the period begins, the number of hours of staffing provided by permanent staff in this period is calculated for each ward. For this, the number of planned staff who are not unexpectedly absent (i.e. at short notice) is calculated. The chance of being unexpectedly absent can differ between staff types in the model. All these staff are allocated to their home ward to start with. The (absolute) shortfall for each staff type is calculated as required minus allocated hours. Where applicable, the simulation checks which of the registered nurses working are IV-trained (sampled probabilistically).

Similarly the spare hours (hours that could be redeployed to another ward) for each staff type is calculated. This is the allocated minus the required hours, rounded down to the nearest multiple of 'redeployed hours chunk', since staff can only be redeployed for fixed time periods.

On time-step

Next, staff are redeployed within sharing groups (directorates) to attempt to cover shortfalls for each staff type, as shown in FIGURE 2. The shortfall is rounded up or down (depending on the round down bound) to the nearest multiple of 'redeployment chunks'. Requests for extra staff are triggered if the rounded shortfall for that staff type is more than zero, and if either the total shortfall or the staff type shortfall are more than the trigger (6 hours). In order to decide the priority of redeploying extra staff to wards, lists of wards are sorted using the bubble sort algorithm[2] , which

works by comparing the shortfall as a proportion of the requirement (or spare hours as a proportion of requirement) for adjacent wards in the list and swapping them if they are in the wrong order.

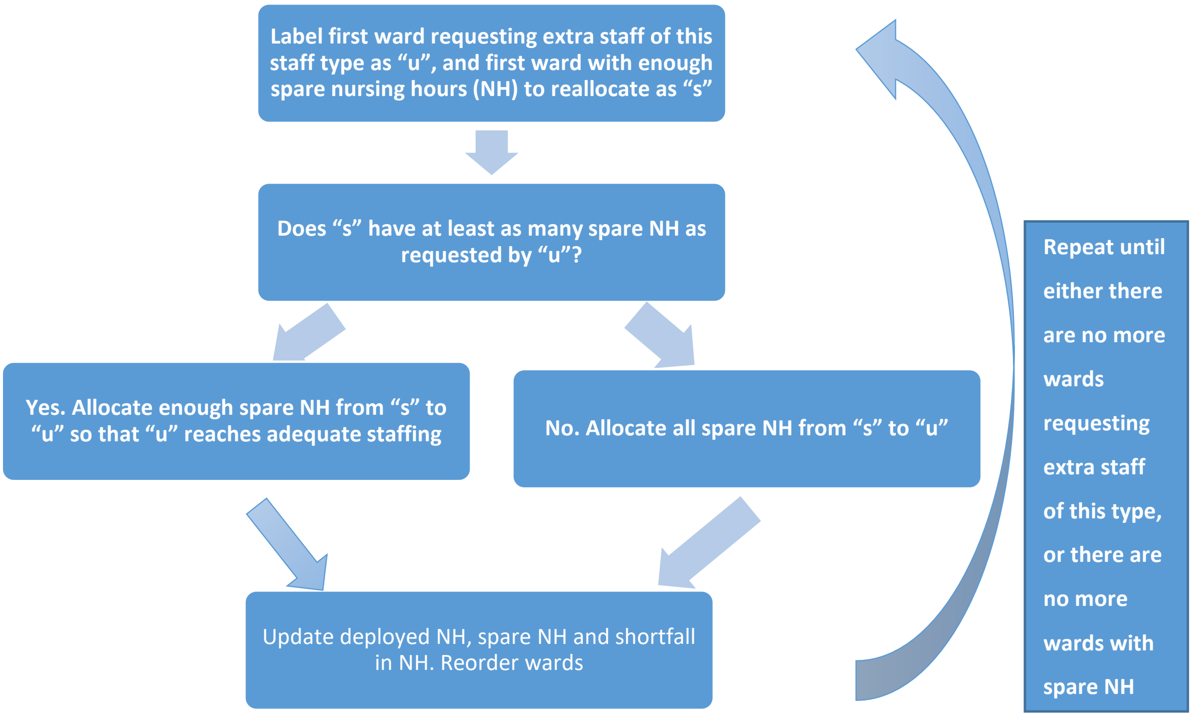

FIGURE 2 Summary process flow of staff redeployment

Following this, for the wards that are still requesting extra staff, for each staff type, first bank and then agency staff are requested, as shown in FIGURE 3. The hours requested are the shortfall rounded up or down (depending on the round down bound) to the nearest multiple of 'external work time'. The probability of a request for temporary staff being fulfilled depends on the staff source, staff type, whether it is a weekday or weekend and the time period.

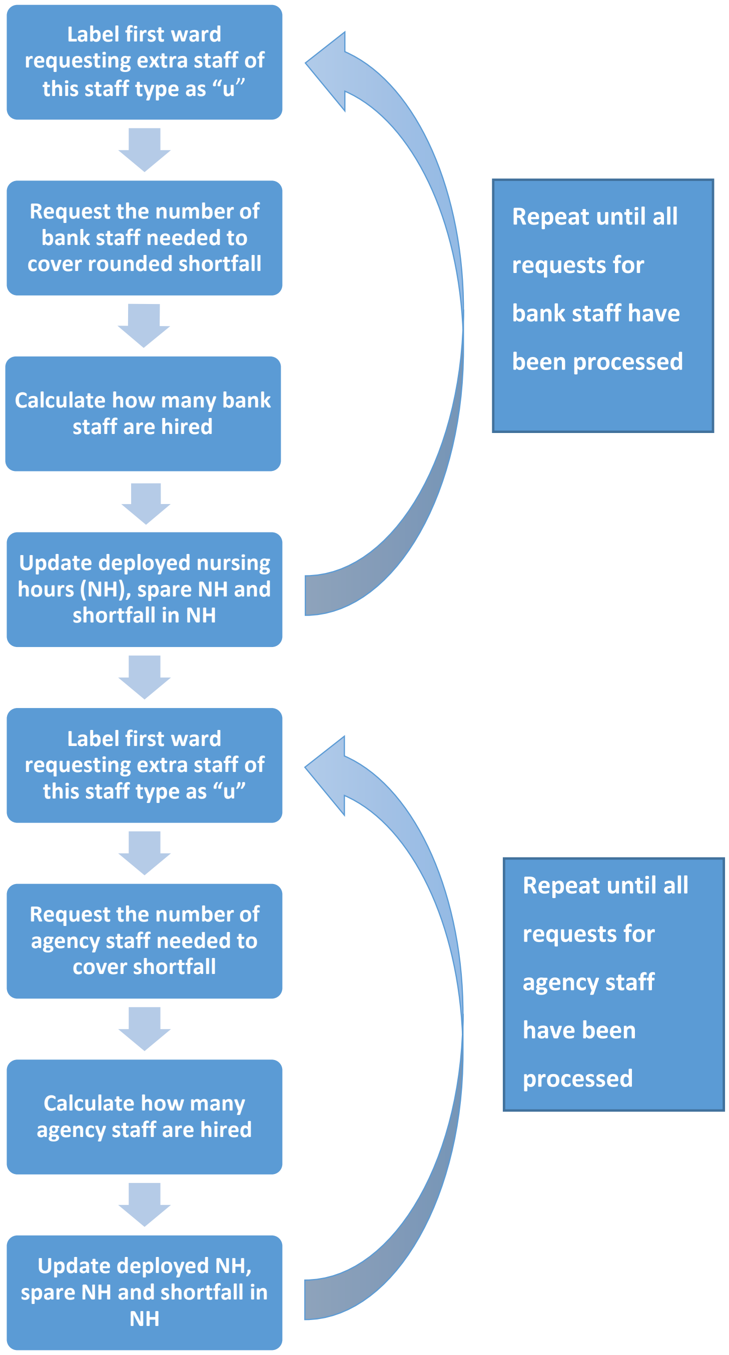

FIGURE 3 Process flow of hiring temporary staff

Then, the effective staffing on each ward, given that redeployed and temporary staff are less efficient than permanent staff, is calculated. The effective shortfall on each ward is calculated.

The staffing adequacy (understaffed, adequately staffed or overstaffed) on each ward according to a range of criteria is assessed, e.g. understaffing can be measured as 'more than 15% under the requirement', 'more than one person short', etc. Depending which staffing adequacy criterion was chosen to be displayed; the state chart for each ward is updated (see Figure 1, main report[1]). The running totals of understaffed, adequately staffed and overstaffed time periods per ward are updated, as well as the charts tracking their relative numbers. The array of the total number of staff deployed on wards from different sources and of different types is updated.

At the end of run (1 year)

At the end of each run, the total permanent staff employed (in WTE) and the number of agency and bank hours worked across the whole hospital are summed up by staff type.

The total yearly costs are calculated. For this, first the costs of employing staff and hiring temporary staff at the standard rates are calculated. Then bonuses for unsocial hours for substantive, bank and agency staff are added on.

The results of the run at both the ward and hospital level are exported to Excel.

Simulation input parameters

Table 1 Hospital (system) input parameters for simulation

Parameter	Description	Value/Distribution	Source/Rationale
**Step length**	Length of time steps. Demand, supply and reallocation calculations happen at the beginning of each time step	6 hours	Six-hour shifts –captures main staffing changes over day
**Round down bound**	Used for dealing with fractional people when requesting permanent staff, internal redeployments or temporary staff. The number of hours is either rounded up or down to the nearest assignment length (four or six hours, see Temporary staff shift length and Redeployed staff shift length), depending on the round down bound. 0 means always round down, 1 means always round up, 0.5 means round to nearest assignment length and if halfway between then round up.<br>e.g. The hours are divided by 6. If the remainder divided by 6 is below the round down bound, the hours are rounded down to the nearest 6, otherwise up to the nearest 6.	0.5	Common way of rounding.
**Multipliers**	The Safer Nursing Care Tool (SNCT) multipliers	For general wards: 0.99,1.39, 1.72, 1.97 and 5.96.<br>For acute admissions units: 1.27, 1.66, 2.08, 2.26 and 5.96.	From the SNCT guidance[3]
**Specials multiplier**	The extra hours of care for a specialed patient	5.4656	Assumes additional 24-hour 1:1 care. Specials multiplier=(24*1.22*7)/37.5
**Standard deviation of multipliers**	Standard deviation of hours per patient day (HPPD) required within an acuity/dependency level	10% of multiplier (0 for specials). Sample from normal distribution	Assumption. Given the lack of data on variability of patient nursing requirements within acuity/dependency levels, we investigated different values in sensitivity analyses.

Parameter	Description	Value/Distribution	Source/Rationale
		truncated at mean +/-2 standard deviations	
**Staffing level**	Rule for setting the establishment from SNCT data	Varied across establishment scenarios - ‘Flexible (low)’, ‘Standard’, ‘High’ or ‘vacancy’ – and across data samples - ‘January’, ‘June’, ‘All’, or ‘Months’.	
**Unanticipated absence chance**	The chance that a registered nurse (RN)/ nursing support worker will be absent at short notice	4% for nursing support workers, 3% for RNs	Assumption. Sensitivity analysis. According to the Office of National Statistics, the total nurse absence rate was 4.5% in 2016/17 [4] but it is not clear how much of this is anticipated, and so can be dealt with by adapting the roster. According to our hospital Trust partners, nursing support worker absence rate is higher than registered nurse absence rate.
**Absence length**	The length of an absence. (Time steps are independent of one another)	6 hours	Half a 12-hour shift. Experts (hospital Trust Principal Investigators [PIs]) agreed reasonable
**Temporary staff shift length**	The length of time temporary staff (bank/agency) are deployed for.	6 hours	Half a 12-hour shift. Experts (hospital Trust PIs) agreed reasonable
**Redeployed staff shift length**	The length of time substantive staff are redeployed to another ward.	4 hours for nursing support workers and 6 hours for registered nurses	Half an 8/12-hour shift. Experts (hospital Trust PIs) agreed reasonable
**Staff type allocation rules**	When there are staffing shortfalls, the order in which different types of additional staff are allocated.	Like-for-like. Bank before agency.	Experts (hospital Trust PIs) advised that these are reasonable general rules (although will not always be true).
**Efficiency**	The productivity of redeployed/temporary staff	Efficiency of redeployed/bank staff – 90%.	Assumption. Recognises that agency staff, who work across different hospitals, will be less familiar with ward processes than

Parameter	Description	Value/Distribution	Source/Rationale
		Efficiency of agency staff – 75%.	redeployed/bank staff who are familiar with hospital but not necessarily with ward.
**Constraint**	Minimum number of registered nurses present on each ward shift.	1 per ward and time step	Assumption. One registered nurse must always be present since there are some tasks only registered nurses can do and nursing support workers require supervision.
**Prioritisation sequence**		prioritise wards with biggest proportion shortfall/ spare (rule number 1)	Assumption. Recognises that will prioritise wards that are most in need for receiving extra staff/ have most spare for providing extra staff. Experts (hospital Trust PIs) agreed reasonable
**Vacancy rate**		0%	Assumption. We also investigated the effect of a 10% vacancy rate in each ward.
**Wards that can lend/ borrow staff**		Share across directorate	Assumption. Recognises that staff are trained to work in particular areas.
**Probability of a request for temporary staff being fulfilled**	Depends on the staff source, staff type, whether it is a weekday or weekend and the time period.	Empirical observed proportions	Based on data on previous-day and same-day staffing requests from hospital Trust B.

Table 2 Ward (agent) input parameters for simulation

PARAMETER	DESCRIPTION	VALUE/DISTRIBUTION
**NUMBER OF PATIENTS**	Each time step and for each ward, the number of patients.	Sampled (with replacement) from observed (empirical) distributions of patient counts at 7am, 1pm, 7pm, 1am for that ward, separately for each day of the week.
**PROBABILITIES OF PATIENTS BEING IN EACH ACUITY/DEPENDENCY LEVEL AND THAT PATIENT REQUIRES SPECIALING**	Each time step and for each ward, the probabilities of patients being in each acuity/dependency level and of patient requiring specialing, e.g. For one shift on one ward, probabilities are Prob(level 0) =0.4, Prob(level 1a)=0.4, Prob(level 1b)=0.2,<br>Prob(level 2) =0, Prob(level 3) =0 and Prob(special)= 0.05.	Sampled with replacement from observed acuity/dependency proportions for that ward and observed specials proportions for that ward.
**PLANNED SKILL MIX**	The planned proportion of registered nurses for the morning, afternoon, evening and night six-hour shifts	The average observed skill mix in each ward for each time period. Note that planned registered nurse/nursing support worker hours are subject to a minimum constraint of one registered nurse and rounded to the nearest six hours
**REQUIRED SKILL MIX**	Of the required staffing each six-hour shift on each ward, how much is required to be done by registered nurses.	The average observed skill mix in each ward for each time period but the required registered nurse/nursing support worker hours are also subject to a minimum constraint of one registered nurse (no rounding).
**PLANNED DISTRIBUTION OF STAFF OVER THE DAY**	The proportion of planned staffing that occurs in each six-hour shift (morning, afternoon, evening and night).	The average observed proportion of worked hours in each time period for each ward.
**REQUIRED DISTRIBUTION OF STAFF OVER THE DAY**	The proportion of daily staffing required in each six-hour shift (morning, afternoon, evening and night).	As for planned distribution of staff over day.

Variability within acuity/dependency levels

We have no information about the shape and variability of nursing requirement distributions within acuity/dependency levels. Even with relatively small variability within acuity/dependency levels (standard deviation set at 10% of the mean) and assuming they follow symmetric distributions, the required staffing for patients in levels 1a, 1b, and 2 would overlap substantially, as shown in *Figure 4* and even more so for a standard deviation at 25% of the mean, as shown in *Figure 5*.

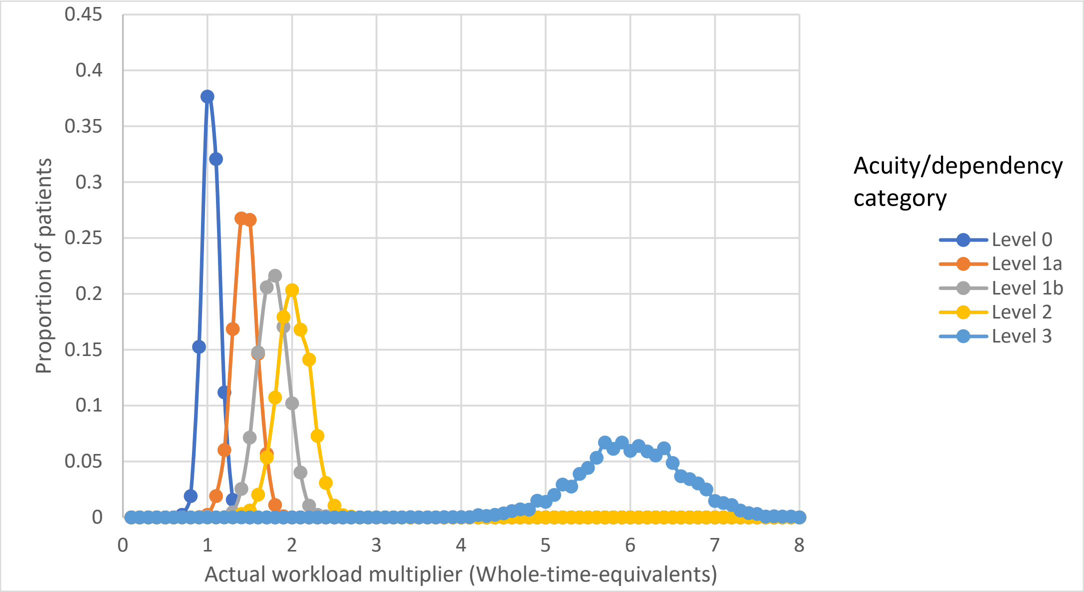

Figure 4 Histogram of required whole-time-equivalents if standard deviations within acuity/dependency levels were 10% of the means (multipliers)

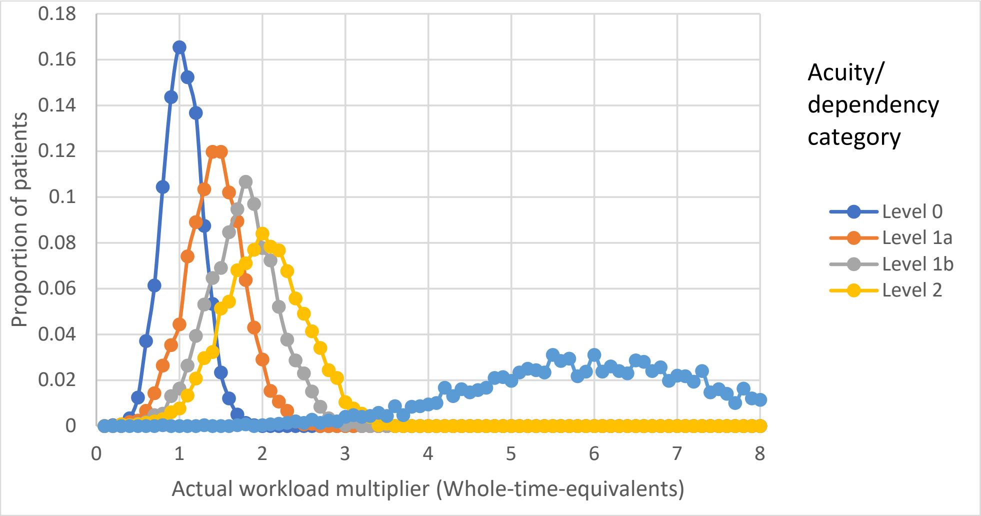

Figure 5 Histogram of required whole-time-equivalents if standard deviations within acuity/dependency levels were 25% of the means (multipliers)

Saville C, *et al. BMJ Qual Saf* 2020; 30:7–16. doi: 10.1136/bmjqs-2019-010569

Staff availability

Table 3 shows the empirical percentages of all requested temporary shifts (both bank and agency) that were filled by bank staff at hospital Trust B, i.e. assumes that bank staff were asked first. Morning shifts are those that start between 7am-1pm, afternoon between 1pm-7pm, evening/night between 7pm-7am. Table 4 shows the percentage of empirical percentages of agency requests that were filled at hospital Trust B. We did not have equivalent data for the other hospital Trusts.

Table 3 Percentage of same-day and previous-day bank requests filled at hospital Trust B

REQUESTS FULFILLED BY BANK STAFF	REGISTERED NURSES	NURSING SUPPORT WORKERS
**WEEKDAY**		
**MORNING**	28%	28%
**AFTERNOON**	10%	9%
**EVENING/NIGHT**	19%	37%
**WEEKEND**		
**MORNING**	32%	31%
**AFTERNOON**	13%	17%
**EVENING/NIGHT**	19%	32%

Table 4 Percentage of same-day and previous-day agency requests filled at hospital Trust B

REQUESTS FULFILLED BY AGENCY STAFF	REGISTERED NURSES	NURSING SUPPORT WORKERS
**WEEKDAY**		
**MORNING**	15%	36%
**AFTERNOON**	9%	5%
**EVENING/NIGHT**	45%	44%
**WEEKEND**		
**MORNING**	19%	43%
**AFTERNOON**	14%	14%
**EVENING/NIGHT**	37%	39%

Saville C, *et al. BMJ Qual Saf* 2020; 30:7–16. doi: 10.1136/bmjqs-2019-010569

Simulation outputs

Table 5 Performance measures used to compare simulation scenarios

Key performance measures	Description
Percentage understaffed patient shifts	We say a shift is 'understaffed' if the total effective staffing (i.e. accounting for the lower efficiencies of temporary and redeployed staff) is less than 85% of the estimated requirement according to the Safer Nursing Care Tool (SNCT) for the shift. This allows a buffer of 15 per cent either side of the estimated requirement within which staffing is considered adequate, as is also done in the RAFAELA tool.[5] We consider patient shifts rather than shifts in order to estimate how many understaffed shifts individual patients are exposed to. We express the understaffed *patient* shifts (the sum of the occupancies at the start of each understaffed shift) as a percentage of the total patient shifts (the sum of the occupancies at the start of each shift).
Cost per patient day	The annual staffing cost divided by the number of patient shifts, multiplied by four (in our model there are four six-hour shifts in a day).
**Other performance measures**	
Ward establishments	The number of staff employed on each ward under a particular scenario. Measured in whole time equivalents.
Percentage of hours worked by redeployed/bank/ agency staff	
Percentage overstaffed patient shifts	We say a shift is 'overstaffed' if the total effective staffing (i.e. accounting for the lower efficiencies of temporary and redeployed staff) is more than 15% over the estimated requirement according to the SNCT for the shift.
Percentage of patient shifts with no intravenous-trained registered nurse	Used only for the scenario looking at intravenous (IV)-trained nurses.
**Alternative understaffing criteria**	
Percentage patient shifts with absolute understaffing	'Absolute understaffing' means the total effective staffing is less than the estimated requirement for the shift.
Percentage patient shifts with at least one person short	'At least one person short' means the total effective staffing is at least six hours below the estimated requirement for the shift.
Percentage patient shifts with registered nurse shortfall	'Registered nurse shortfall' means the effective registered nurse staffing is less than 85% of the estimated registered nurse requirement for the shift.
Percentage patient shifts with nursing support worker shortfall	'Nursing support worker shortfall' means the effective nursing support worker staffing is less than 85% of the estimated nursing support worker requirement for the shift.
Percentage patient shifts with both	'Both' means there is both 'registered nurse shortfall' and 'nursing support worker shortfall' on this shift.
Percentage patient shifts with either	'Either' means there is either 'registered nurse shortfall 'or 'nursing support worker shortfall' on this shift.

Table 6 Simulation data validity

CATEGORY	ITEM	TECHNIQUE(S) USED	JUSTIFICATION FOR TECHNIQUE USED	RESULT/ CONCLUSION	CONFIDENCE IN RESULT
**COLLECTING AND MAINTAINING DATA**	SNCT data	Pilot study<br><br>Inter-rater reliability	For finding any issues.<br><br>For checking how well SNCT ratings agree between responders	It was feasible to collect data on busy wards. Cognitive testing showed staffing adequacy questions were understood as intended.<br><br>At the shift level, based on the calculation of total staffing scores using the multipliers there was agreement within +/- 5% of the joint mean on 95% of occasions. (In total 847 patients were rated independently by two raters across 81 shifts.)	Medium
**TESTING RELATIONSHIPS WITHIN DATA**	Checking aggregations correct	Checking numbers of records before and after merging data tables.	Standard way	Numbers were consistent.	High
**SCREENING FOR OUTLIERS AND DETERMINING WHETHER CORRECT (AND DECIDING WHETHER TO INCLUDE IN MODEL)**	Weighted average multipliers<br><br>Patient numbers	Screening: points outside the mean plus or minus three standard deviations for weighted average multipliers. Whether correct: compared against ward change dates and known unusual times of year e.g. Christmas and New Year. Deciding whether to include in model: removed because likely to be data error.<br><br>Screening: Looked at periods with low numbers of patients. Whether correct: compared against ward change dates and known unusual times of year e.g. Christmas and New Year. Deciding whether to include in model: removed low numbers of patients during ward change periods but kept if due to strange time of year e.g. Christmas	Standard technique combined with practical insights	Outliers dealt with appropriately	High

Table 7 Conceptual model validity

CATEGORY	ITEM	TECHNIQUE(S) USED	DETAILS	JUSTIFICATION FOR TECHNIQUE USED	RESULT/CONCLUSION	CONFIDENCE IN RESULT
**ASSUMPTIONS**	Independence of requirements between time periods	Face validity	team meeting, steering group	No data available on this	No data on how time periods may depend on one another, so best we can do.	Low
	Independence of requirements in different wards	Face validity	team meeting, steering group	No data available on this	No reason to suppose wards do interact.	Medium
	Order of redeploying staff to wards	Face validity	team meeting, steering group. Expert opinion on how would prioritise redeploying staff	Since we want to generalise results, we are interested in a sensible likely method rather than capturing the details of how each hospital/directorate currently does this.	After a few iterations, agreed internal redeployment, then bank then agency, and like-for-like substitutions.	Medium-high
	Temporary staff shift length	Face validity	team meeting, steering group.	Since we want generalisable results, we want a sensible approximation	Agreed that using multiples of 6-hours would capture most shift patterns	Medium-high
	Redeployed staff shift length	Face validity	team meeting, steering group.	Since we want generalisable results, we want a sensible approximation	Agreed that using multiples of 6-hours for registered nurses and 4-hours for nursing support workers would capture most shift patterns	Medium-high
	Absence length	Face validity	team meeting, steering group.	Since we want generalisable results, we want a sensible approximation	Agreed that using multiples of 6-hours would capture most shift patterns	Medium-high
**MODEL REPRESENTATION**	Rounding logic	Structured walkthrough	With PG and JJ, showed Excel sheet with step-by step calculations for translating baseline staffing in WTE into registered nurse and nursing support worker staffing per shift	To agree sensible logic for rounding staffing levels with other quantitative researchers	Agreed after a few iterations	High
	Structure, detail and aggregate	Face validation	Showed logical steps of (various	To build credibility and gain expert opinion from	Trust PIs and steering group agreed in general with the	High

	relationships (wards and hospitals), logic, mathematical (e.g. calculating requirements) and causal relationships (e.g. understaffing leads to redeploying staff)	iterations of) model in presentation s to steering group and to hospital Trust PIs (Principal Investigators)	steering group and Trust PIs	conceptual model, and suggested some changes (such as how staffing requirements are calculated for specials) that were incorporated in subsequent iterations of model development

Table 8 Operational validity of simulation

CATEGORY/ ITEM	TECHNIQUE(S) USED	DETAILS	JUSTIFICATION FOR TECHNIQUE USED	RESULT/CONCLUSION	CONFIDENCE IN RESULT
**ASSUMPTIONS**	Sensitivity analyses with design of experiments	2^k factorial design	Used to test how much outputs are influenced by parameters we do not have data for.	Apart from the round down bound, the other factors made less than a 5-percentage point difference to the percentage understaffed patient shifts and less than a £0.50 difference to cost per patient day. We agreed with our Trust partners that rounding to the nearest whole person rather than always rounding up was most realistic.	High
**INPUT-OUTPUT RELATIONSHIPS**	Face validity	Circulated around team	Building credibility and expert opinion	No scenario simulation results were identified as unlikely/surprising	High
**INTERNAL VALIDITY**	Replications	Determining stochastic variability using 10 replications of 1 year	Standard technique	The between-year variation at hospital level is low enough that alternative scenarios have significantly different results.	High

Table 9 **Computer model verification**

CATEGORY/ITEM	TECHNIQUE(S) USED	JUSTIFICATION FOR TECHNIQUE USED	RESULT/ CONCLUSION	CONFIDENCE IN RESULT
**ERROR-FREE SIMULATION LANGUAGE, SIMULATION LANGUAGE PROPERLY IMPLEMENTED**	Trace	Used a simulation language so simulation debugger picked up most errors in the language. CS checked for problems with e.g. array sizes by tracing through calculations.	Yes	High
**MODEL PROGRAMMED CORRECTLY**	Animation, operational graphics, trace	Animation of numbers of staff from each source used to show whether redeployments working correctly.<br><br>Operational graphics of understaffed/overstaffed wards over time used to quickly spot errors, e.g. all wards understaffed.<br><br>Put in actual staffing levels, occupancy and acuities. Compared understaffed shifts output of simulation with actual understaffed shifts.	Yes	High
**PSEUDO RANDOM NUMBER GENERATOR PROPERLY IMPLEMENTED**	Trace	Used standard Anylogic in-built RNG so has already been tested for correctness, so just needed to check that sampling was working correctly in our model.	Yes	High

Table 10 **Overall simulation verification and validity**

EVALUATION AREA	OVERALL CONCLUSION	JUSTIFICATION FOR OVERALL CONCLUSION	CONFIDENCE IN CONCLUSION
**CONCEPTUAL MODEL VALIDITY**	The model is a sufficiently accurate representation of the real world.	Model developed in partnership with nursing experts from four hospital Trusts. (Not high because some of the data needed to check assumptions are not readily available.)	Medium - high
**COMPUTER MODEL VERIFICATION**	Computer model programmed correctly	Range of techniques used throughout model development process	High
**OPERATIONAL VALIDITY**	The simulation model results are similar enough between runs to be able to draw conclusions.	Face validity was tested and variability between run results was low.	High
**DATA VALIDITY**	Data sufficiently valid – improving data validity further unlikely to impact conclusions	Outliers were removed, although it is hard to distinguish between valid and invalid outliers. Inter-rater-reliability between SNCT ratings was high.	Medium - High

References

1. Griffiths P, Saville C, Ball J, et al. Identifying nurse-staffing requirements using the Safer Nursing Care Tool. Modelling the costs and consequences of real world application to address variation in patient need on hospital wards (In Press). *Health Services and Delivery Research Journal* 2019 doi: https://www.journalslibrary.nihr.ac.uk/programmes/hsdr/1419421/#/
2. Knuth DE. The Art of Computer Programming, Vol. 3: Sorting and Searching. 2nd Edition ed: Addison-Wesley 1981.
3. The Shelford group. Safer Nursing Care Tool Implementation Resource Pack: The Shelford Group, 2014.
4. Moberly T. Sickness absence rates across the NHS. *BMJ* 2018;361 doi: https://doi.org/10.1136/bmj.k2210
5. Rauhala A, Fagerström L. Determining optimal nursing intensity: the RAFAELA method. *Journal of Advanced Nursing* 2004;45(4):351-59. doi: 10.1046/j.1365-2648.2003.02918.x
