# Supplementary material for: Costs and consequences of using average demand to plan baseline nurse staffing levels: a computer simulation study
Source: BMJ Qual Saf. 2020 Mar 26;30(1):7–16. doi: 10.1136/bmjqs-2019-010569 (PMC7788209; doi:10.1136/bmjqs-2019-010569)
Supplement: Supplementary data [file bmjqs-2019-010569supp002.pdf]

Appendix Table: Temporary/redeployed staff use

Baseline staffing	Low	Standard	High	Low	Standard	High	Low	Standard	High
**Flexible staffing option**	**Worked hours**			**Redeployed**			**Bank / Agency**		
	**N**	**N**	**N**	**%**	**%**	**%**	**%**	**%**	**%**
**Unlimited availability**	4584595	5157822	5444865	0.2%	0.5%	0.8%	29.9%	14.9%	10.9%
**Higher availability**	5480128	5616905	5788361	0.2%	0.5%	0.8%	24.9%	12.0%	8.6%
**Empirical availability**	5107618	5432740	5647155	0.2%	0.6%	0.9%	16.3%	7.3%	5.2%
**No temporary staff or redeployments**	3837858	4780113	5158532	0.0%	0.0%	0.0%	0.0%	0.0%	0.0%
